# Circadian optimisation of irinotecan and oxaliplatin efficacy in mice with Glasgow osteosarcoma

**DOI:** 10.1038/sj.bjc.6600168

**Published:** 2002-03-18

**Authors:** T G Granda, R-M D'Attino, E Filipski, P Vrignaud, C Garufi, E Terzoli, M-C Bissery, F Lévi

**Affiliations:** INSERM E-0118 Chronothérapeutique des cancers and Université Paris XI, Institut du Cancer et d'Immunogénétique, Hôpital Paul Brousse, 14, av. Paul Vaillant Couturier, 94800 Villejuif, France; Aventis Pharma SA, Vitry Sur Seine, France; Oncologia Medica Complementare, Regina Elena Institute, Rome, Italy

**Keywords:** circadian, irinotecan, oxaliplatin, Glasgow osteosarcoma

## Abstract

The relevance of circadian rhythms in irinotecan and oxaliplatin tolerability was investigated with regard to antitumour activity. Mice bearing Glasgow osteosarcoma (GOS) received single agent irinotecan (50 or 60 mg kg^−1^ per day) or oxaliplatin (4 or 5.25 mg kg^−1^ per day) at one of six dosing times expressed in hours after light onset (3, 7, 11, 15, 19 or 23 hours after light onset). Irinotecan (50 mg kg^−1^ per day) and oxaliplatin (4 or 5.25 mg kg^−1^ per day) were given 1 min apart at 7 or 15 hours after light onset, or at their respective times of best tolerability (7 hours after light onset for irinotecan and 15 hours after light onset for oxaliplatin) or worst tolerability (15 hours after light onset for irinotecan and 7 hours after light onset for oxaliplatin). Tumour growth rate was nearly halved and per cent increase in estimated life span (% ILS) was – doubled in the mice receiving irinotecan at 7 hours after light onset as compared to 15 hours after light onset (*P*<0.05). Results of similar magnitude were obtained with oxaliplatin for both endpoints, yet with 7 hours after light onset corresponding to least efficacy and 15 hours after light onset to best efficacy (*P*<0.05). Irinotecan addition to oxaliplatin proved therapeutic benefit only if the schedule consisted of irinotecan administration at 7 hours after light onset and oxaliplatin delivery at 15 hours after light onset, i.e. when both drugs were given near their respective ‘best’ circadian times. These would correspond to the middle of the night for irinotecan and the middle of the day for oxaliplatin in humans.

*British Journal of Cancer* (2002) **86**, 999–1005. DOI: 10.1038/sj/bjc/6600168 www.bjcancer.com

© 2002 Cancer Research UK

## 

Irinotecan and oxaliplatin are two recently available drugs which have largely improved the efficacy of chemotherapy against metastatic colorectal cancer (Cunningham *et al*, 1998; Rougier *et al*, 1998; De Gramont *et al*, 2000; Douillart *et al*, 2000; Giacchetti *et al*, 2000). Gastrointestinal and haematologic toxicities are dose limiting for both agents, while irinotecan can also cause asthenia and oxaliplatin can produce peripheral sensory neuropathy.

Experiments in mice have indicated that the tolerability and the efficacy of both agents varied significantly as a function of drug dosing time, as a result of the rhythms which modulate cell metabolism and proliferation along the 24 h time scale (Lévi, 1997). Cellular circadian rhythms are generated by interacting molecular loops, involving at least eight specific genes (Balsalobre *et al*, 1998; Dunlap, 1999; Van der Horst *et al*, 1999; Whitmore and Sassone-Corsi, 1999). They are coordinated by the suprachiasmatic nuclei, a group of neurons located at the floor of the hypothalamus (Klein *et al*, 1991). The regular alternation of light and darkness over 24 h synchronises mammalian circadian rhythms and constitute a reference for predicting the times of peak and trough of cellular metabolism and proliferation.

As a consequence of this circadian time structure, the haematologic and intestinal toxicities of oxaliplatin were significantly reduced by the administration of this drug near the middle of darkness as compared to midlight in mice (Boughattas *et al*, 1989; Li *et al*, 1998). The clinical relevance of this finding was subsequently demonstrated in randomised trials involving cancer patients treated with oxaliplatin as a single agent or combined with 5-fluorouracil and leucovorin (Caussanel *et al*, 1990; Lévi *et al*, 1997, 2000). Indeed, the chronomodulated administration of oxaliplatin, with peak delivery at 16:00 h resulted in fewer patients presenting with neutropenia, diarrhoea or peripheral sensory neuropathy as compared to constant rate infusion or chronomodulated administrations with peak delivery rates occurring 9 or 12 h apart.

Three studies have also indicated that the tolerability of camptothecin derivatives – irinotecan and 9-aminocamptothecin – varied as a function of time of administration in mice (Filipski *et al*, 1997; Ohdo *et al*, 1997; Kirichenko and Rich, 1999). Body weight loss as well as intestinal and haematological toxicities were least following treatment delivery in the second half of the rest span of mice. A pilot clinical study has suggested that a chronomodulated infusion of irinotecan could improve the tolerability of this drug, an issue which is currently being investigated in a randomized trial (Giacchetti *et al*, 1997).

We have studied the relationship between the circadian rhythm in the tolerability of irinotecan and that of oxaliplatin and the anticancer efficacy of these drugs in mice. We also have examined the activity of their combination along the circadian time scale, since several reports have suggested synergistic activity (Scheithauer *et al*, 1999; Wasserman *et al*, 1999; Goldwasser *et al*, 2000).

## MATERIALS AND METHODS

### Study design

Four experiments were conducted in a total number of 400 male B6D2F_1_ mice bearing Glasgow Osteosarcoma (GOS) at a palpable stage. For this reason, all treatments were applied 5 days after tumour transplantation. Injections were repeated daily for 4 consecutive days (d5–8) (Table 1

**Table 1:**
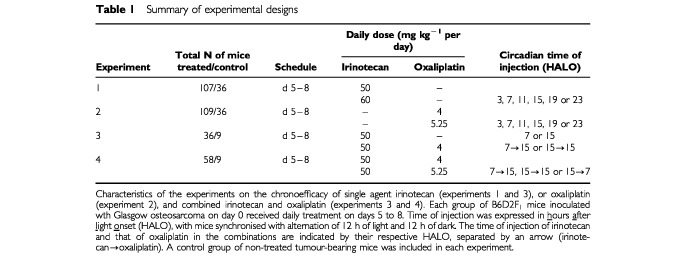
Summary of experimental designs

).

The role of dosing time upon single agent irinotecan or oxaliplatin activity was investigated in experiments 1 and 2 respectively. All the mice were synchronised with an alternation of 12 h of light (L) and 12 h of darkness (D) (LD 12 : 12). Injection times were expressed in hours after light onset (HALO). Each drug was given daily at either dose level at one of six dosing times, staggered by 4 h. Controls received NaCl 0.9% (experiment 1) or 5% glucose (experiment 2) at each dosing time. Experiments 3 and 4 tested the role of dosing time of irinotecan – oxaliplatin combination. In experiment 3, single agent irinotecan was given at 7 or 15 HALO. Combined irinotecan and oxaliplatin were injected 1 min apart at either dosing time, selected on the basis of the results from experiments 1 and 2. In experiment 4, both drugs were given at 15 HALO (1 min apart), or irinotecan was given at 7 HALO and oxaliplatin at 15 HALO, near their respective ‘best times’ as single agents, or irinotecan was administered at 15 HALO and oxaliplatin at 7 HALO, near their respective ‘worst time’ as single agents.

### Drugs

Irinotecan (solution for intravenous (i.v.) injection, 20 mg ml^−1^) was kindly provided by Aventis Pharma (Montrouge, France). The drug was diluted in 0.9% sodium chloride on each study day, prior to injections. Oxaliplatin (solution for i.v. injection, 5 mg ml^−1^) was kindly provided by Sanofi-Synthélabo (Montpellier, France). The drug was diluted in 5% glucose on each study day prior to injections. The final drug solutions were injected i.v. into the right retro-orbital venous sinus (10 ml kg^−1^ of body weight). Mice received an average of 0.28 ml of solution at each injection. Irinotecan and oxaliplatin doses were selected on the basis of preliminary experiments in this model at our laboratory (D'Attino *et al*, unpublished results). For experiment 1, single agent irinotecan dose was 50 mg kg^−1^ per day (total dose of 200 mg kg^−1^) or 60 mg kg^−1^ per day (total of 240 mg kg^−1^), which represents a 20% dose escalation. For experiment 2, single agent oxaliplatin dose was 4 mg kg^−1^ per day (total dose of 16 mg kg^−1^) or 5.25 mg kg^−1^ per day (total dose of 21 mg kg^−1^), which represents a 31% dose escalation. For experiments 3 and 4, the doses were 50 mg kg^−1^ per day for irinotecan and 4 or 5.25 mg kg^−1^ per day for oxaliplatin.

### Animals and synchronisation

All experiments were carried out in accordance with the guidelines approved by the UKCCCR.

Male B6D2F_1_ mice, 6 to 7 weeks of age, were purchased from IFFA-Credo (L'Arbesle, France). They were housed two per cage with water and food *ad libitum*, and allocated to one of the several treatment groups planned for each experiment. Cages from each group were placed on a different shelf of an autonomous chronobiological animal facility (ESI-Flufrance, Arcueil, France). Each facility has six soundproof, temperature-controlled compartments, each having its own programmable lighting regimen, corresponding to six different circadian stages each one located 4 h apart. Each compartment was constantly provided with filtered air delivered at an adjusted rate.

Six circadian times were tested during the light span, when the animals are usually at rest (3, 7 or 11 HALO) or during darkness, when the animals are usually active (15, 19 or 23 HALO).

Rectal temperature was measured with a rectal probe after a minimum of 2 weeks' synchronisation in groups of mice from each compartment.

### Tumour

Glasgow osteosarcoma (GOS), a tumour known to display intermediate sensitivity to both irinotecan and oxaliplatin was provided by the Research Centre of Aventis Pharma (Vitry sur Seine, France). The tumour was maintained in C57BL/6 female mice over 6 weeks of age and passaged every 2 weeks as bilateral subcutaneous (s.c.) implants in donor female C57BL/6 mice until the lower tumour weight reached 700 mg.

Donor mice were sacrificed, their tumours were removed, placed into Hank's balanced salt solution and dissected into fragments measuring 3×3 mm using a grill scaled to these values. Recipient experimental mice were transplanted with one tumour fragment in each flank, using a trocar.

Mice with tumour weight reaching 2000 mg along the course of the study were sacrificed for ethical reasons and were considered as dead from tumour progression on this date.

### Study endpoints

Body weight was measured with a precision balance and tumour weight was determined using a caliper, three times a week:


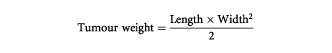


Mortality was recorded daily for up to 60 days.

Time to reach a mean tumour weight of 2 g was considered as an estimate of survival. The median day to reach this endpoint was used as median survival time estimate (MSTE) in order to compute the percentage of Increase in Life Span (%ILS) as described elsewhere (Tampellini *et al*, 1998).

### Statistical analysis

Rectal temperature, tumour weight and body weight changes were analysed with analysis of variance (ANOVA). Differences in tumour weight were analysed in data truncated on the study day preceding the death of 50% of the mice in one treatment group. Survival curves were drawn according to Kaplan–Meier, and differences in survival were tested using the log-rank method. Proportions of mice with tumour ⩽2 g were compared using the χ^2^ test. All standard statistics were performed using SPSS for Windows software. The statistical significance of circadian rhythmicity was further documented by cosinor analysis (Nelson *et al*, 1979).

## RESULTS

### Synchronisation of mice

Mean rectal temperature of tumour bearing mice (±s.e.m.) varied from 35.1±0.1°C in the light span up to 38.6±0.1°C in the dark span. ANOVA validated significant differences as a function of sampling time (*P*<0.001). Cosinor analysis revealed a significant circadian rhythm for each experiment (*P*<0.01). The acrophase (time of maximum in fitted curve) was localised at 18.28 HALO (95% C.L.,±68 min), close to the middle of the dark span.

### Tumour growth and survival of control mice

All the control mice died from tumour progression between 11 days and 19 days after tumour inoculation. No significant difference in tumour growth or survival was found as a function of the circadian time of tumour inoculation, whether the data were examined separately for each experiment or pooled. Overall, the MSTE of control mice ranged from 13 days (experiment 3) to 15 days (experiment 4) without any statistically significant difference between the four experiments.

### Single agent irinotecan (experiment 1)

#### Lethal toxicity

A single mouse receiving 50 mg kg^−1^ per day of irinotecan died from toxicity immediately after the fourth dose, at 15 HALO. Early mortality was encountered within the first two treatment days in the mice receiving 60 mg kg^−1^ per day. No death was found following drug dosing at 7 HALO. Conversely, mortality rate was 30% in the mice treated at 19 HALO and 55.5% in those injected at 23 HALO (χ^2^=9.7; d.f.=5; *P*=0.08).

#### Body weight change

Mean body weight reached a nadir on the fourth treatment day, without any dose effect (*F* from ANOVA=0.7, N.S.). Conversely, dosing time significantly influenced body weight loss at each dose level (two-way ANOVA, F_dose_=0.6, NS; F_time_=11.2, *P*<0.0001). Body weight loss at nadir was nil in the mice treated at 7 or 11 HALO, whatever the dose level, and largest in those treated at 19 HALO (7.5±2.1% with 50 mg kg^−1^ per day and 14.6±2.8% with 60 mg kg^−1^ per day).

#### Tumour growth

Irinotecan produced no partial or complete tumour regression, but rather slowed down tumour growth as compared to controls. Tumour size was compared as a function of dose and dosing time before mortality from tumour progression. Tumour growth was slower in the mice treated at 7 HALO as compared to those injected at 23 HALO (Table 2

**Table 2:**
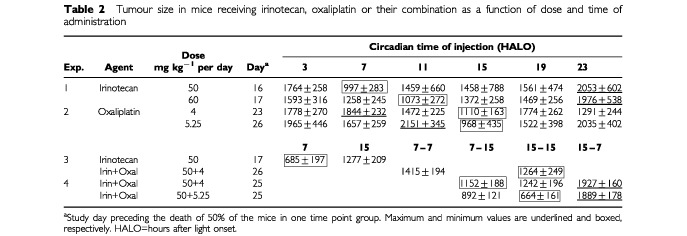
Tumour size in mice receiving irinotecan, oxaliplatin or their combination as a function of dose and time of administration

). Death from tumour progression occurred in the mice receiving 50 mg kg^−1^ per day between the 14th and the 26th day after tumour inoculation (Figure 1A

**Figure 1:**
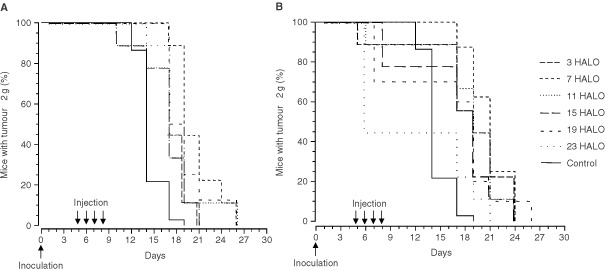
Percentage of GOS-bearing B6D2F_1_ male mice with tumours less than 2 g. Animals received four consecutive daily i.v. injections of irinotecan at one of six dosing times, expressed in hours after light onset (HALO). Two dose levels were tested: 50 mg kg^−1^ per day (**A**) or 60 mg kg^−1^ per day (**B**). Differences in curves of estimated survival (time to reach tumour weight of 2 g) as a function of dosing time were tested by log rank (*P*=0.19, **A** and *P*<0.05 **B**).

). At this dose level, MSTE was 19 days at 7 HALO and 16 days for all the other treatment times, with corresponding %ILS of 58.3 and 21.4%. Death from tumour progression occurred in the mice receiving 60 mg kg^−1^ per day between the 17th and the 26th day after tumour inoculation. The survival curves differed significantly as a function of treatment time for the higher dose and for the pooled data at both dose levels (*P* from log rank <0.05). At the higher dose level, MSTE ranged from 6 days at 23 HALO (%ILS, −50%) to 21 days at 7 HALO (%ILS, +75%).

### Single agent oxaliplatin (experiment 2)

#### Lethal toxicity

No mouse died from toxicity following the administration of 4 mg kg^−1^ per day. Conversely, 12.7% of toxic deaths were encountered in the mice treated with 5.25 mg kg^−1^ per day. In this latter group, the mortality rate was 22.2% in the mice injected at 3 HALO, as compared to 12.5% in those treated at 15 HALO and 11.1% in the animals receiving the drug at 19 HALO (χ^2^=0.9; NS).

#### Body weight change

Mean body weight loss reached a nadir 5 or 12 days after treatment onset in the mice treated with 4 or 5.25 mg kg^−1^ per day of oxaliplatin respectively. The average maximum weight loss was 5.9±0.7% in the mice receiving 4 mg kg^−1^ per day as compared to 9.2±1.0% in those treated with 5.25 mg kg^−1^ per day. Body weight loss at nadir differed significantly as a function of both dose (*F* from ANOVA=16.6, *P*<0.0001) and dosing time at each dose level (*F* from ANOVA=6, *P*<0.0001). Maximum weight loss following the administration of 5.25 mg kg^−1^ per day was largest in the mice treated during the light span (12.6±2.5% at 3 HALO, 10.0±2.2% at 7 HALO) as compared to those given the drug during the dark span (4.3±2.1% at 15 HALO and 5.4±1.3% at 19 HALO).

#### Tumour growth

**Figure 2:**
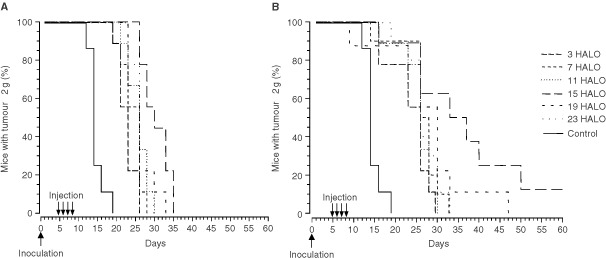
Percentage of GOS-bearing B6D2F_1_ male mice with tumours less than 2 g. Animals received four consecutive daily i.v. injections of oxaliplatin at one of six dosing times, expressed in hours after light onset (HALO). Two dose levels were tested: 4 mg kg^−1^ per day (**A**) or 5.25 mg kg^−1^ per day (**B**). Curves of estimated survival (time to reach tumour weight of 2 g) differed as a function of dosing time for each dose level (*P* from log rank <0.001, **A** and *P*=0.018, **B**).

Tumour growth was slower in the mice receiving oxaliplatin at 15 HALO as compared to those treated at 7 or 11 HALO (Table 2). All the mice which received 4 mg kg^−1^ per day of oxaliplatin were sacrificed for tumour reaching 2 g between 19 and 35 days after tumour inoculation. The mice receiving 5.25 mg kg^−1^ per day were killed for this reason between 19 and 47 days after tumour inoculation, except one cured animal. MSTE ranged from 23 to 33 days as a function of dose (*P* from Log Rank=0.04). Survival curves also differed significantly as a function of dosing time (Figure 2

). Thus, at the lower dose level, MSTE was 30 days in the mice treated at 15 HALO (%ILS, 114%) as compared to 23 days in those injected at 3 HALO (%ILS, 64%) (Figure 2A). At the higher dose level, MSTE was 33 days following dosing at 15 HALO (%ILS, 136%) and 26 days in the mice given the drug at 3 HALO (%ILS, 86%). The single cured mouse had received 5.25 mg kg^−1^ per day at 15 HALO and it was still alive on day 90 (Figure 2B).

### Irinotecan-oxaliplatin combination (experiments 3 and 4)

#### Lethal toxicity

No mortality was found in experiment 3. In experiment 4, lethal toxicity was limited to the combination involving 5.25 mg kg^−1^ per day of oxaliplatin and varied as a function of treatment time and schedule. Mortality rate ranged from 33.3% in the mice given both drugs at 15 HALO as compared to 9.1% in those receiving irinotecan at 15 HALO then oxaliplatin at 7 HALO and 8.3% of the mice treated with irinotecan at 7 HALO then oxaliplatin at 15 HALO (χ^2^=0.9; N.S.).

#### Body weight change

In experiment 3, mean body weight loss reached a nadir 10 days after tumour inoculation. The average maximum weight loss (±s.e.m.) was 15±0.9% with both drugs given at 7 HALO and 10±1.8% if they had been given at 15 HALO (*F* from ANOVA=22.7, *P*<0.001). In experiment 4, mean body weight loss reached a nadir 11 days after tumour inoculation. The average maximum weight loss was 16±3.4% in the mice injected at 15 HALO with irinotecan and the higher oxaliplatin dose, as compared to 10±0.9% in the animals given irinotecan at 7 HALO and the lower oxaliplatin dose at 15 HALO.

#### Tumour growth

In experiment 3, the combination delayed by 9 days the time to reach 2 g in 50% of the mice from one treatment group as compared to irinotecan alone (Table 2). Tumour growth inhibition with single agent irinotecan was nearly twice as large at 7 HALO as compared to 15 HALO. The irinotecan–oxaliplatin combination seemed to be more active when both drugs were given at 15 HALO rather than at 7 HALO (Table 2).

The administration of oxaliplatin 1 min after that of irinotecan improved estimated survival as compared to single agent irinotecan, whether both agents were given at 7 HALO (%ILS, 115% for the combination *vs* 62% for single agent irinotecan) or at 15 HALO (%ILS, 154% for the combination *vs* 46.1% for single agent irinotecan).

**Figure 3:**
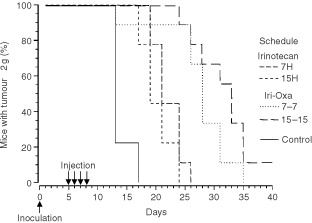
Percentage of GOS-bearing B6D2F_1_ male mice with tumours less than 2 g. Animals received four consecutive daily i.v. injections of irinotecan (50 mg kg^−1^ per day) or irinotecan-oxaliplatin combination (50 and 4 mg kg^−1^ per day, respectively) at one of two dosing times (7 or 15 HALO), expressed in hours after light onset. Prolongation of estimated survival (time to reach tumour weight of 2 g) with combination varied significantly as a function of dosing time (*P* from log rank <0.005).

Tumour growth retardation varied significantly as a function of combination dosing time (*P* from log rank <0.005) (Figure 3

). Thus, the delivery of both drugs 1 min apart resulted in longer MSTE and %ILS in the mice treated at 15 HALO as compared to those injected at 7 HALO (MSTE, 33 *vs* 28 days; %ILS, 154% *vs* 115%).

In experiment 4, maximum tumour reduction was achieved with the highest dose of oxaliplatin (5.25 mg kg^−1^ per day) combined with irinotecan. Oxaliplatin was more effective at 15 HALO, irrespective of dose (Table 2). The administration of irinotecan at 15 HALO and that of oxaliplatin at 7 HALO was clearly less effective than any other combination schedule.

**Figure 4:**
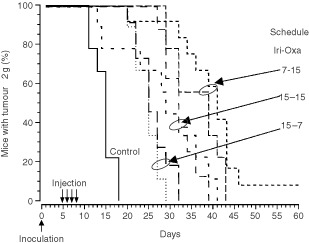
Percentage of GOS-bearing B6D2F_1_ male mice with tumours less than 2 g. Animals received four consecutive daily i.v. injections of two combinations of 50 mg kg^−1^ per day of irinotecan and 4 mg kg^−1^ per day of oxaliplatin (light line) or 5.25 mg kg^−1^ per day (thick line). Three schedules of administration were tested: both drugs at 15 HALO, or irinotecan at 7 HALO and oxaliplatin at 15 HALO, or irinotecan at 15 HALO and oxaliplatin at 7 HALO. Curves estimated survival (time to reach tumour weight of 2 g) differed as a function of dosing time, (*P* from log rank <0.005).

Irinotecan–oxaliplatin combination proved of no benefit as compared to single agent oxaliplatin, if irinotecan was given at 15 HALO and oxaliplatin at 7 HALO (MSTE, 25 days; %ILS, 77%). The most effective regimen consisted in the administration of irinotecan at 7 HALO and oxaliplatin at 15 HALO (Figure 4

). The MSTE of the mice receiving this latter schedule was 39 days if the oxaliplatin dose was 4 mg kg^−1^ per day (%ILS, 160%) and 41 days after an oxaliplatin dose of 5.25 mg kg^−1^ per day (%ILS, 173%). Statistically significant differences were found as a function of irinotecan–oxaliplatin circadian schedule (*P* from log rank <0.005).

## DISCUSSION

The antitumour efficacy of irinotecan and oxaliplatin, both as single agents and combined, varied significantly as a function of circadian dosing time in tumour-bearing mice.

Glasgow osteosarcoma was chosen since it displayed intermediate sensitivity to both drugs, a property which is also shared by human colorectal cancer and which is useful for studying the benefit from irinotecan–oxaliplatin combination.

The tolerability of irinotecan, as assessed with body weight change and survival, was best in the second half of the rest span in GOS-bearing mice, a result similar to that previously reported in non-tumour bearing animals (Filipski *et al*, 1997; Ohdo *et al*, 1997). Pseudocholinergic shock was responsible for immediate mortality in the mice receiving 60 mg kg^−1^ per day. This toxic effect was encountered in 30% of the mice treated at 19 HALO, in 55% of those given the drug at 23 HALO and in none of the animals treated at 7 HALO. As a result, the toxicity rhythm largely contributed to the significant dosing time-related differences in survival. On the other hand, tumour growth inhibition with the non toxic dose (50 mg kg^−1^ per day) was nearly twice as large in the mice treated at 7 HALO also, as compared to those receiving the drug during the dark span, both in experiments 1 and 3. As a result, the %ILS was nearly twice as large in the mice receiving a toxic or a non-toxic dose at 7 HALO as compared to 19 HALO. This suggests that GOS susceptibility to irinotecan displays a circadian pattern. Thus, the full dose-efficacy relationship could be explored meaningfully only at 7 HALO, an issue which was beyond the scope of the current study.

In the second part of the study a strong rhythm in oxaliplatin efficacy was found at either dose level. No toxic death was produced by 4 mg kg^−1^ per day of oxaliplatin. For the higher dose (5.25 mg kg^−1^ per day) lethal toxicity was 12.7%. Both lethal toxicity and body weight loss were nearly twice as high in the mice treated at early to mid light as compared to near mid dark. The tolerability rhythm was similar to that reported with a single dose of this drug (Li *et al*, 1998; Boughattas *et al*, 1989). In the present study, tumour growth inhibition and %ILS were also largest at 15 HALO and poorest at 3 or 7 HALO. Thus, the rhythm in oxaliplatin efficacy overlapped that in tolerability, with best therapeutic index with drug dosing at 15 HALO.

We subsequently explored the role of dose interval and circadian time for irinotecan–oxaliplatin combination. In order to avoid irinotecan early mortality, this agent was only given at a dose of 50 mg kg^−1^ per day. Irinotecan was combined with either oxaliplatin dose (4 or 5.25 mg kg^−1^ per day×4 days). In experiment 3, the administration of both drugs 1 min apart significantly slowed tumour growth and prolonged %ILS as compared to single agent irinotecan. The most effective schedule consisted in the delivery of both drugs at 15 HALO. In experiment 4, this latter schedule was compared to the administration of both drugs near their respective ‘best’ times (7 HALO for irinotecan and 15 HALO for oxaliplatin) or ‘worst’ times (15 and 7 HALO). This latter schedule was clearly less active with regard to tumour growth inhibition than both other treatment regimens, indicating the need to administer oxaliplatin at 15 HALO. Nevertheless, nearly 30% of the mice receiving both drugs at 15 HALO died from lethal toxicity as compared to less than 10% with the other schedules. This supports the administration of irinotecan at 7 HALO and oxaliplatin at 15 HALO as the best tolerated and the most active combination schedule.

**Figure 5:**
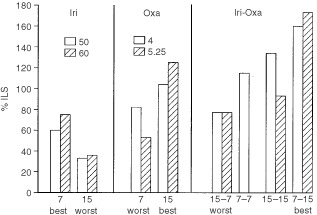
Summarised results. Best and worst efficacy schedules for single agent irinotecan and oxaliplatin and for their combination, as assessed with percentage of increase of estimated life span (%ILS).

We compared the increase in survival estimate (%ILS) from the irinotecan–oxaliplatin combination schedules with that from each single agent (Figure 5

). The combination was significantly more effective than any single drug only if irinotecan was given in the middle of the rest span (7 HALO) and oxaliplatin near the middle of the activity span (15 HALO).

The results support that a synergistic activity of these drugs requires their administration near their respective ‘best’ circadian times. They are in line with the coincidence between the time of best efficacy and that of best tolerability which was recently shown for single agent doxorubicin, docetaxel or vinorelbine and for docetaxel–doxorubicin combination (Tampellini *et al*, 1998; Filipski *et al*, 1999; Granda *et al*, 2001).

The results from the present study support the chronomodulated infusion of irinotecan and oxaliplatin in patients with metastatic colorectal cancer with respective peak delivery rate near 5:00 and 16:00 h respectively, in order to optimize both tolerability and efficacy of this treatment schedule.
